# Counter-Irritants1Read before the twenty-first regular meeting of the Missouri Valley Veterinary Medical Association.

**Published:** 1899-12

**Authors:** J. A. Sloan

**Affiliations:** St. Joseph, Mo.


### COUNTER-IRRITANTS,[1]

By J. A. Sloan, M.D.V.,
ST. JOSEPH, MO.

A COUNTER-IRRITANT is a drug or process by means of which an irritation is produced in one or more parts of the body with the view of relieving one already existing in another part, and for the purpose of strengthening some part or to hasten the reparative process of some part. As a remedial process counter-irritants occupy a great and useful sphere in veterinary practice. Their use in human medicine is of great antiquity, for they were mentioned by ancient writers on medicine; but there are few of our people to-day who would consent to be "marked" with a hot iron for the alleviation of pain or the cure of disease. True, the hot iron is still used as a counter-irritant, but

[1] Read before the twenty-first regular meeting of the Missouri Valley Veterinary Medical Association.

only to promote a slight congestion of the skin, and when so used is first heated in boiling water and then lightly applied; or, if heated to a red heat, the iron is slowly waved back and forth near the skin.

Counter-irritants are commonly divided into two classes: internal and external. Internal counter-irritants are known as revulsives or derivatives, and may be defined as remedies "which, by producing a modified action in some organ or tissue, overcomes the morbid condition of some other organ or tissue." A very familiar example is the administration of a drastic purgative to relieve active cerebral hyperæmia or a powerful diuretic to deplete the system in dropsical conditions. External counter-irritants are those which are used upon the surface of the body, and are the more common in use. These only will be considered as within the scope of this article. They may be defined as "substances used externally to produce a reflex influence on a part more or less remote from the site of application." It is true that these drugs are sometimes directly applied to the part to be relieved or strengthened; but, as the subject of counter-irritants is too extensive to be discussed in all of its phases, we will omit this one as of minor importance.

This second class is again divided according to severity of action: Those of mild action, or rubefacients, which only cause a congestion of the skin. If their action is continued they may become vesicants, in which vesicles or blisters are formed. This is an eructate or outpouring of serum into the lower layers of the epithelium and liquefaction of epithelial cells beneath the horny layer, which causes vesicles to appear to be between the epidermis and derma. If contact is still continued a third subdivision is reached called escharotics; these involve destruction of all tissues to which the irritant is applied and the formation of a slough. A fourth subdivision, still more severe in action, is pustulants, which give rise to pustules on the parts to which they are applied, as the orifices of the cutaneous glands. A fifth subdivision is setons and rowels, which are inserted through openings in the skin or the injection of an irritant substance beneath the skin, and are used to set up a deep-seated, long-continued, and extensive inflammation.

Another classification: (1) Those used externally for the cure of internal diseases; these cure by revulsion, relieve pain, produce diaphoresis, and arrest forming disease; the counter-irritants commonly used to produce these effects are hot applications, cantharides, and liniments. (2) Those used in the treatment of lamenesses, etc. These promote a cure by increasing the activity of the superficial parts and of the circulation. They tend to destroy the elasticity of the skin because of a decided thickening, and prevent the use of the leg or obstruct motion because of the soreness of the skin. They also, when not too severe, cause hyperæmia of the superficial parts and anæmia of the deeper structures; but when severe, the inflammation becomes deep-seated, which is proven by the fact that an increased amount of fibrous tissue has been found in the deeper parts of a leg years after the application of a severe blister or firing iron. Again, when animals are treated they are given a rest from work, which is often an aid in curing the disease. The counter-irritants commonly used in the order of their importance are cantharides, mercuric iodide or biniodide, actual cautery, setons, and liniments.

To make cantharides blister, take eight parts of lard or vaseline, heat to 120° F., and stir in one part of the pulverized cantharides. As excessive heat tends to destroy the active principle, or cantharidin, a complex animal substance easily broken up into simpler compounds, the vehicle should not be heated very low. If applied over a very large surface a proportion of 1 : 16 is sufficiently strong, and even then it causes irritation of the kidneys and excessive diuresis, which may continue for a week.

Biniodide of mercury is also mixed with lard or vaseline in a proportion of 1 part to 4, 6, or 8. It is indicated in such cases as require a more severe treatment than fly blister, and will diminish enlargements of the soft and hard structures. These two blisters, or the so-called mixed blister, should never be used together, for the action of cantharides is quicker, and the exudate it causes washes off the biniodide before it has been absorbed, thereby causing a loss of material. Sometimes a little of the biniodide may be used every night until the skin is blistered, when it should be stopped until the skin is healed.

In ordinary firing line-firing is preferable, and if neatly done need not "mark" an animal much more than point-firing. If the client objects to the patient being marked, fire lightly and apply a strong blister; but if no objection is made, it is better to fire well and apply a light blister. In either case, after two days shampoo with soap and warm water and grease with lard. Point-firing is little better than a good blister; and while it may satisfy the scruples of a client, it does not begin to compare with a good line-firing for effectiveness. The only advantage of firing over blisters is that the irritation thus set up is deeper seated, longer continued, and more severe.

The technique of applying counter-irritants is, briefly, clip the hair, because it in part protects the skin from the action of the drug. Brush the clipped surface thoroughly to get rid of dirt. The brushing also increases the activity of the skin, and consequently the rapidity of the absorption of the drug. Apply the blister, using only what the skin will absorb readily, and rub briskly with the hand for a few minutes. If the exudate is likely to run down to parts not treated, smear with vaseline to prevent loss of hair, for the exudate is itself an irritant and will set up an inflammation. Tie the patient short by backing in a stall for about eight hours, by which time the severe pain will have passed off. For two days tie the patient so that he cannot lie down or in any way spread the blister to other parts. Then shampoo thoroughly the parts treated with castile soap and warm water for half an hour, dry, and smear with lard. Apply the lard daily for three days, shampoo again, and continue this treatment for ten days, when all the scabs will be removed and no more formed. The patient will be ready for work on the fourteenth day, or may be turned out to grass in season. If it is necessary to get the patient to work as soon as possible, apply a more severe treatment than would otherwise be necessary, and after two days decrease and limit the action of the irritant by the continual application of hot stupes. This treatment should be begun immediately after the first shampooing. The stupes may be wetted with an astringent solution to hasten the process. By this treatment the scabs may all be removed and the patient ready for work in eight or ten days from the application of the blister. It is necessary to exercise care and judgment in the application of counter-irritants to obtain the "golden mean" of the right amount of inflammation and avoid all danger of sloughing. This is obtained by experience and quick perception of the peculiarities of the patient. In treating lameness it is not always desirable to induce the formation of a thick scab by the application of a blister. To recapitulate: If it is necessary to produce considerable and continued inflammation, use the actual cautery; and if a lesser amount of irritation will produce the desired effect, use a blister; but in either case, in two days after the application, curative measures should be adopted to reduce or check this superficial inflammation, instead of allowing the formation of a thick scab, which will require two or three weeks to slough off. All that should be required of a blister is to set up an inflammation which will increase the activity of the parts, and either relieve the morbid condition and hasten repair or hurry the inflammatory action to a natural termination as seen in anchylosis. When repair is aided the curative action is due to an increased activity of the circulation, the elimination and absorption of morbid products, and the restoration of the natural functions. When the morbid process terminates in anchylosis the increased circulation aids in the formation of new tissues and its final calcification.

*Discussion.*

Dr. Stewart: In opening this discussion I must congratulate the essayist upon his happy condensation of so broad a subject into concise statements, and especially the clearness with which he has given the technique of application of the counter-irritants in diseases of the extremities. It is perhaps in this field more than any other that counter-irritants are employed. The paper does not deal particularly with the application of counter-irritants for the relief of internal diseases, yet it is probable that this was the original use of this class of agents. They were used for the relief of pain of a neuralgic character, as well as the results of inflammation, particularly in pleurisy. The paper does not exhaustively deal with the theories as to how counter-irritants produce their effect. It is held by some that the pain the irritation produces on the surface of the body at one point will lessen the arterial injection and nerve sensibility in some other part of the body, and it is this peculiar power which indicates their employment. It is a matter of common record that in cases of pleurisy in the first or dry stage a sharp irritant applied over the chest-wall is promptly followed by relief of the pain, and sometimes by the entire subsidence of the disease processes. Professor Williams, of Edinburgh, is reported to have stated that counter-irritants were of little value to relieve pain or congestion or to modify chronic inflammations; and Finlay Dun records some experiments by Professor McCall which tend to show that powerful counter-irritation of the skin induces inflammation of the skin and subcutaneous tissues, but that perceptible changes do not occur in the deeper parts. Many practitioners believe that a counter-irritant applied over the throat region will relieve the distress in acute pharyngitis and laryngitis, and it was my custom to apply a blister over enlarged lymphatic glands consequent upon an attack of strangles which did not suppurate in the usual length of time. The blister seemingly hastened suppuration and resolution.

The essayist probably indicated the most important point in connection with the firing and blistering for relief of chronic inflammations when he stated that a long rest was an important factor. It has been held by some good practitioners that rest alone, without the counter-irritation, would effect a cure. In relation to the comparative value of line-firing and puncture-firing, I will say that with my limited experience the best results were obtained from the puncture-firing; but it has been a question in my mind whether or not the good results were not due to the direct irritation of the deeper parts produced by the puncture, rather than from the cauterization of the skin, it being doubtful about producing cauterization in the deeper parts. Perhaps others will discuss this point more fully.

Dr. Moore: Relative to the choice between line-firing and puncture-firing, I will say that it has been my custom to employ the line-firing where I desired to secure the influence of a counter-irritant in diseases of the soft parts, but in diseases of the bones I have preferred to use puncture-firing. I am quite inclined to believe that by puncturing of the periosteum in cases of bony growths, such as spavins and ring-bones, the tension on the periosteum is lessened, resulting in relief and cure.

The essayist has indicated that the combination of cantharides and biniodide of mercury is a bad one, and as I have used this combination for several years with seemingly good results, I feel called upon to defend it. However, I would not employ these active agents in so large a proportion as indicated by the essayist. I use one drachm of cantharides and one-half drachm of mercuric iodide to the ounce of vaseline, and have found this a very reliable and certain blister. In no case have I found that the cantharides acted first and the serum produced washed off the slower-acting mercury. While I am aware that oftentimes the cantharides on the market is very inferior in quality, and that failure to secure results from cantharides preparations may be fairly ascribed to this, I have sought to overcome this possible difficulty by combining the two agents in the same blister, and they have always acted admirably for me.

Dr. Milnes: There was one point in relation to the employment of the actual cautery which the essayist did not touch upon, which seems to me to be quite important, and that is the age of the animal to be fired. My experience has taught me that we should be very careful in firing young animals, such as weanlings and yearlings. They are very susceptible to the action of counter-irritants, and great damage may be done if they are not cautiously applied.

Dr. Ernst: I would like to ask the essayist his opinion as to whether in the application of the firing-iron and blister over a spavin or ring-bone if he thinks that the inflammation is confined to the skin and subcutaneous tissues, or extends to the deeper parts?

Dr. Sloan: I believe it is possible to do a job of line-firing that will extend clear to the bone.

It is probable that killing the animal so early as the third day, in the experiments made by Dr. McCall, that the tissue changes in the deeper structures would not be noted, as proliferation of connective-tissue cells would not take place so early. Had the animals lived longer, changes deeper down would doubtless have been noted.

Dr. Stewart: In the experiments alluded to the visible evidence of inflammation, such as congestion of vessels and exudate into the tissues, did not extend below the subcutaneous connective tissues. There was not observable any vascular changes in the deeper structures.

I had hoped some one would take up the problem of modifying the circulation in the deeper parts by means of irritation of the surface. This is certainly quite an interesting phase of the application of counter-irritation. It is well known to general practitioners that the stimulation of the skin by moist heat determines increased circulation in this structure which will relieve internal congestion. If an animal suffering from congestion of the lungs or other internal organs be wrapped in blankets wrung out of water as hot as the animal can bear, and then further covered with blankets and impervious covers, the internal congestion will be promptly relieved. A like result may be obtained by a thorough friction of the surface with some stimulating liniment, and the body wrapped in blankets. I feel that this is quite an important phase of the application of counter-irritants, and one not so much employed as it should be.

A letter received from a party in Maine inquires if an injured leg is fired whether the other corresponding limb, which is sound, should be also fired, and as it seems to be the custom to fire both limbs if one is fired, will some one give a good reason for it?

Dr. Sloan: It has been my custom to fire or blister both limbs if I fire or blister one, so that the animal may be inclined to stand on both alike, as it is well known that laminitis may be developed if the weight is borne almost entirely on one limb.

Dr. Bennett: The horse-owners of Kentucky have great faith in firing and blistering in diseases of the horse's limbs, and are quite heroic in their application, not caring particularly if there be some blemishing so that the animal goes sound.

I have had most excellent results with cantharides, and I prepare my blister by dissolving one drachm of cantharides in six drachms of olive oil, the material being heated over a water-bath for half an hour and stirred constantly. The active element may deposit a little, but it is readily incorporated again in the oil by stirring. I have employed this blister in cases of tumors of the withers, which you know terminate in fistulæ, and in other parts with most excellent results. As a blister to be applied over the joints and body-growths, I usually employ a mixture of red iodide of mercury and vaseline, one part to six. When I have used the firing-iron I have also applied the cantharides blister to reinforce its action. It is quite important to rub the blister in thoroughly in order to secure the best results.

The Germans are great advocates of firing, and of deep firing. I remember to have seen a case come into the hospital very lame from strain of the suspensory ligament, and he was fired very deep, almost into the ligaments, and a blister applied. In four days he was standing on the limb, and on the eighth day went out of the hospital sound.

Dr. Stewart: I wish to inquire if the last speaker would have us to understand that the horse simply went out free from lameness or that the wounds from firing had also healed?

Dr. Bennett: The wound was not entirely healed, but the horse went perfectly sound.

Dr. Stewart: I believe it is important to emphasize the relation of the cantharides to the element in which it is prepared as a blister. The cantharidin, or active principle, is not freely soluble in vaseline, but is quite soluble in lard and oils, and for cantharides to be most active as a blister the cantharidin must be thoroughly dissolved. The parts to which it is applied should also be properly prepared to get the best results. They should be clean and dry, water interfering with the action of the cantharidin. Vaseline is the best medium for suspending and evenly subdividing the mercuric iodide, as it gravitates to the bottom in the lard or oil, and hence is unevenly distributed through the mixture.

Dr. Milnes: In making cantharides blister I made it a rule to buy the best quality of cantharides from a reliable house, and to make the ointment with what is known as simple cerate, dissolving the lard and wax over a water-bath, and stirring in the cantharides and continuing to stir it occasionally for an hour or more until the cantharidin was dissolved. Some benzoin was added to prevent rancidity. This form of ointment was always solid and stable in all temperatures, and was a very reliable blister in my hands. I would like to ask Dr. Bennett if the olive-oil preparation was not inclined to run over the parts below where applied.

Dr. Bennett: The olive oil makes a liquid blister, and I prefer the liquid blister to the blister in the form of ointment. The blister is thoroughly rubbed into the parts, and there is no excess to run upon the parts below. Of course, I took precautions to anoint the structures below to prevent the discharges from the blisters taking the hair off below the part where the blister was applied.

